# A 3D Freehand Ultrasound System for Multi-view Reconstructions from Sparse 2D Scanning Planes

**DOI:** 10.1186/1475-925X-10-7

**Published:** 2011-01-20

**Authors:** Honggang Yu, Marios S Pattichis, Carla Agurto, M Beth Goens

**Affiliations:** 1 Department of Electrical and Computer Engineering, University of New Mexico, Albuquerque, NM 87131, USA; 2 The Children's Hospital Heart Center, Department of Pediatrics, University of New Mexico, Albuquerque, NM 87131, USA

## Abstract

**Background:**

A significant limitation of existing 3D ultrasound systems comes from the fact that the majority of them work with fixed acquisition geometries. As a result, the users have very limited control over the geometry of the 2D scanning planes.

**Methods:**

We present a low-cost and flexible ultrasound imaging system that integrates several image processing components to allow for 3D reconstructions from limited numbers of 2D image planes and multiple acoustic views. Our approach is based on a 3D freehand ultrasound system that allows users to control the 2D acquisition imaging using conventional 2D probes.

For reliable performance, we develop new methods for image segmentation and robust multi-view registration. We first present a new hybrid geometric level-set approach that provides reliable segmentation performance with relatively simple initializations and minimum edge leakage. Optimization of the segmentation model parameters and its effect on performance is carefully discussed. Second, using the segmented images, a new coarse to fine automatic multi-view registration method is introduced. The approach uses a 3D Hotelling transform to initialize an optimization search. Then, the fine scale feature-based registration is performed using a robust, non-linear least squares algorithm. The robustness of the multi-view registration system allows for accurate 3D reconstructions from sparse 2D image planes.

**Results:**

Volume measurements from multi-view 3D reconstructions are found to be consistently and significantly more accurate than measurements from single view reconstructions. The volume error of multi-view reconstruction is measured to be less than 5% of the true volume. We show that volume reconstruction accuracy is a function of the total number of 2D image planes and the number of views for calibrated phantom. In clinical in-vivo cardiac experiments, we show that volume estimates of the left ventricle from multi-view reconstructions are found to be in better agreement with clinical measures than measures from single view reconstructions.

**Conclusions:**

Multi-view 3D reconstruction from sparse 2D freehand B-mode images leads to more accurate volume quantification compared to single view systems. The flexibility and low-cost of the proposed system allow for fine control of the image acquisition planes for optimal 3D reconstructions from multiple views.

## Background

There is a strong interest in developing effective 3D ultrasound imaging systems in ultrasonography. The basic advantage of 3D systems is that they enable us to provide quantitative measurements of different organs without assuming simplified geometrical models associated with conventional 2D systems. Here, our focus is on developing a 3D ultrasound system for accurate 3D reconstructions from arbitrary scanning geometries (freehand) that are validated on calibrated 3D targets.

Most research in 3D echocardiography is focused on the use of 3D probes (volume-mode probes) where 3D volume is imaged directly from a single probe position. This approach simplifies the reconstruction and visualization of the 3D data set since the geometry of acquired slices is known and real-time 3D reconstruction is possible. This approach does not allow for fine control of the location of the 2D planes.

Clinically, 3D probes have not been widely adopted. The overwhelming majority of ultrasound exams are still using standard 2D ultrasound probes. Routine clinical diagnosis is still depended on acquiring optimal 2D acoustic views. On the other hand, 3D ultrasound can be used by non-experts to avoid the need for training on how to acquire optimal 2D views [1]. In current clinical practice, 3D datasets are often communicated to expert readers that will then have to extract optimal 2D views that are needed for documenting clinical diagnosis.

In this paper, we discuss the design of a 3D freehand ultrasound system built from standard 2D ultrasound machine (see Figurs 1, 2). This flexible, very low cost approach allows for experts control over the acquisition geometry and meets the clinical examination protocol. While the development of 3D freehand ultrasound is certainly not new [2-7], our focus here is quite different. We are primarily interested in developing reliable image processing methods that can be used to demonstrate:

**Figure 1:**
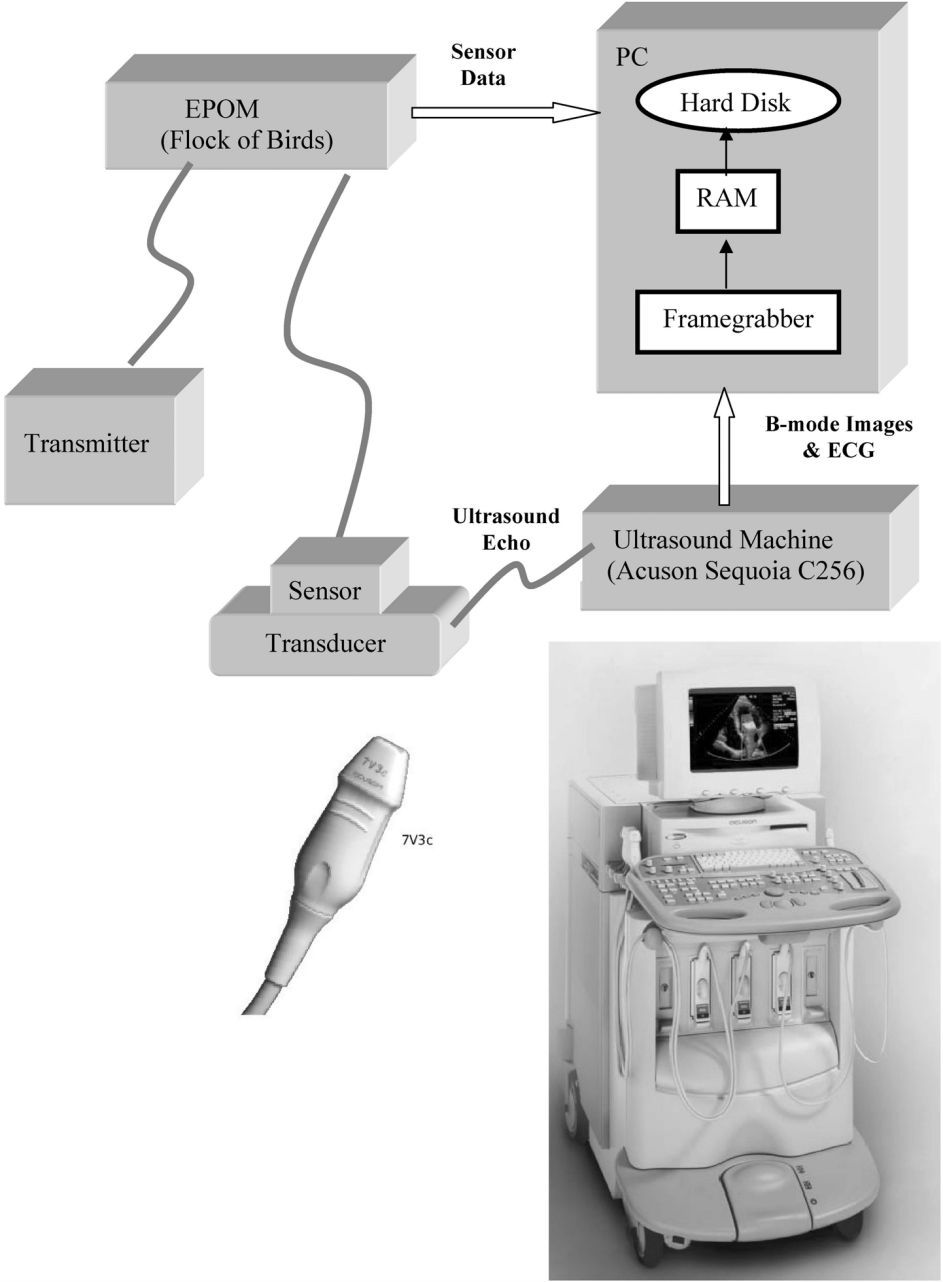
**3D freehand ultrasound prototype system**. The prototype system allows clinicians to optimize the ultrasound acquisition geometry.

**Figure 2:**
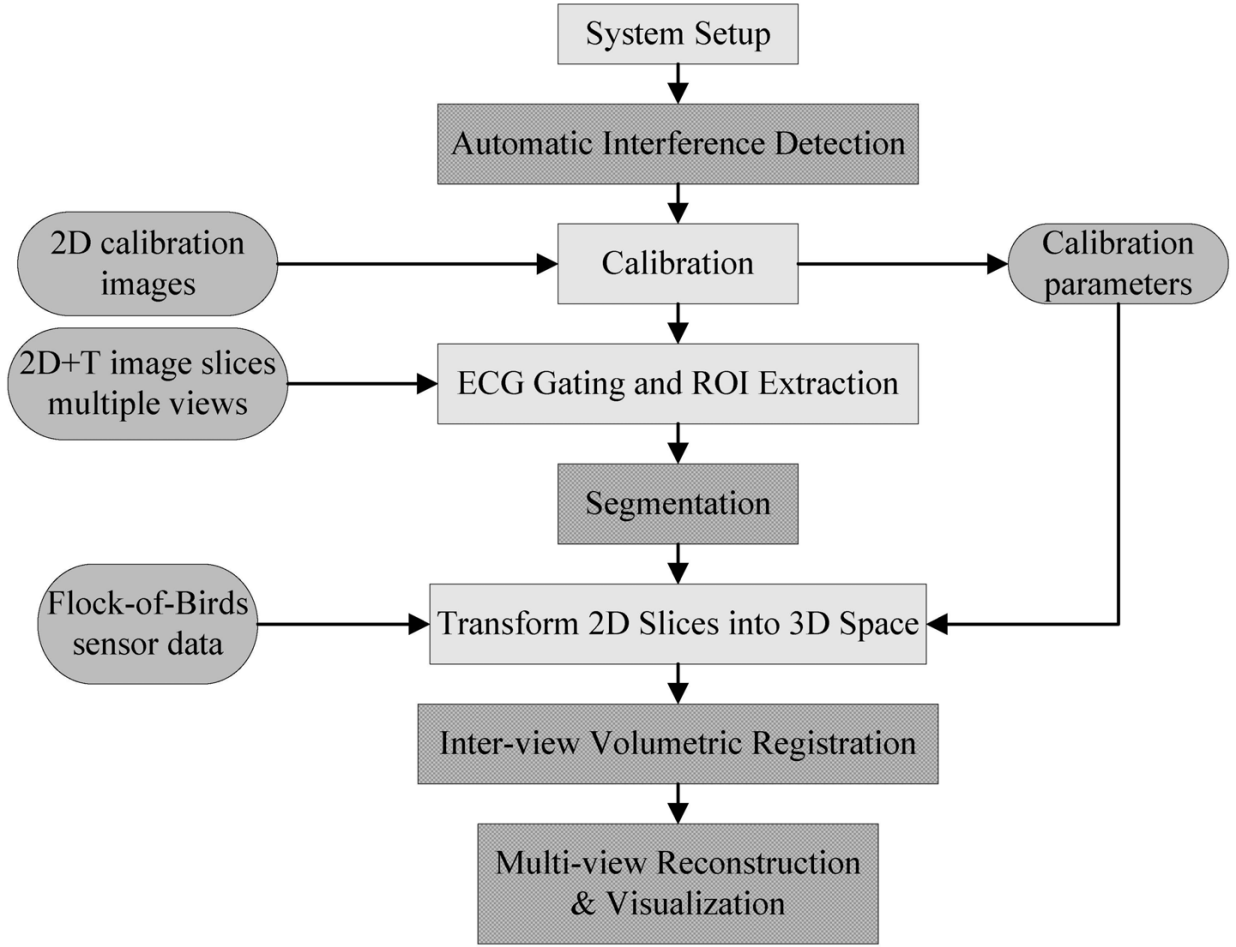
**Software flow-chart for multi-view reconstructions from freehand 2D images**. The software system allows us to measure the effects of interference, the use of a new hybrid segmentation and multi-view reconstruction with automated feature-based registration.

• ***Robust system components:***

Several required image processing system components (see Figure 2) are often treated separately in the literature. In this paper, we address several system issues for reliable performance (see preliminary results in [8-10]). Successful 3D reconstruction in the system begins with reliable electromagnetic interference detection for accurate 3D position and orientation sensing. It also requires accurate 2D to 3D calibration. A hybrid active contour segmentation and parameter optimization is used to develop a robust segmentation method. It is important to note that we segment each 2D plane independently. Due to sparse sampling, 3D segmentation methods are not applicable here. Following segmentation, a robust, coarse to fine, multi-view registration method is used for registering multiple 3D volumes. Here, the registration is based on the 3D geometric shape, and does not depend on the varied gray-scale intensities of different view acquisitions.

• ***Reconstructions from sparse acquisition geometries:***

We are particularly interested in quantifying reconstruction error as a function of the number of acquired ultrasound image planes. The use of a limited number of planes can achieve acceptable 3D accuracy and speed up the clinical examination. It also allows fast screening of normal cases. In our system, 2D images are acquired from different acoustic windows during routine clinical diagnosis. The sparsely sampled 2D images are automatically registered into 3D volumes as opposed to the use of registering densely sampled 3D images in 3D real-time echocardiography.

• ***Multi-view reconstructions:***

There is relatively limited research on the use of multiple views with automated registration. Yet, a large number of imaging artefacts are associated with single view ultrasound image acquisition. As an example, the presence of shadows due to ribs and lungs can significantly limit our imaging capability [11]. Furthermore, anatomical structures produce weak reflections or no reflections when they are parallel to the ultrasound beam. In such cases, almost no echo energy is reflected back to the transducer. These limitations can be addressed through the use of multiple acoustic windows and views where the ultrasound beam can propagate behind obstructions while imaging organ interfaces at the directions that are not parallel to the ultrasound beam.

In our 3D freehand ultrasound prototype system, an electromagnetic position and orientation measurement device (EPOM) is attached on a conventional 2D clinic ultrasound probe (see Figure 1). This allows 3D reconstruction from arbitrary sampling geometries and multiple acoustic windows. It provides a simple hardware system that allows for great flexibility in choosing suitable acoustic windows according to clinical practice. Once a region of interest has been identified, a freehand 3D system allows the experts to take very dense samples around the abnormality from an appropriate acoustic window and view, providing very accurate reconstructions in the region of interest.

Interest in the use of multiple views for providing 3D reconstructions has been primarily focused on reconstructions of the left ventricle. Legget *et al*. [5] used a 2D freehand scanning protocol and manual registration to combine parasternal and apical windows *in-vitro*. A similar system was reported by Leotta *et al*.[6]. Later, Ye *et al*. [7] used a 3D rotational probe and an electromagnetic spatial locator to combine apical long-axis view and parasternal short-axis view. The reconstructions are fused by features and weighted by the image acquisition geometry. Reconstruction was limited by the lack of automated registration. Good spatial alignment of the dense rotational sweeps between different views was assumed. Long acquisition time for each view (3-4 minutes in [7]) may result in unstable motion of the probe.

For ultrasound image registration, Rohling *et. al*. developed an automatic registration method in gall bladder reconstruction [12]. Six, slightly different sweeps were collected and the first sweep is used as baseline. Spatial compounding was performed by registering the last five sweeps to the first baseline based on the estimation of the cross-correlation of 3D gradient magnitude [12] or the usage of landmarks [13]. A review of cardiac image registration among multiple imaging modalities is available in [14]. As stated in [15], due to the varied image quality associated with cardiac ultrasound images, there are few publications focused on echocardiography image registration [16-18]. Mutual information methods also presented difficulties associated with ultrasound image characteristics [19].

For real-time 3D echocardiography registration, Soler *et al*. [20] used manual marked and segmented meshes of the left ventricle to register two different views from apical window by intensity similarity measure. Grau*et al*. [15] registered parasternal and apical views using phase and orientation similarity measures. Their method relied on the use of manual landmark initialization. Here, we note that effective 3D ultrasound registration cannot be based on image intensity alone due to large intensity variations within the same tissue structures and between the different views.

Unlike prior research based on the use of dense 3D samples, this paper is based on the use of sparse 2D planes. We develop a fully-automated registration without manual initialization. To the best of our knowledge, no such research has been reported in the literature. To achieve reliable registration performance, we use a coarse to fine volumetric registration method. We initialize searching for the global optimal registration parameters using a 3D Hotelling transform to construct a reference frame to coarsely register 3D volumes from different acoustic windows. Then, feature-based high accuracy registration is performed using a robust, non-linear least squares algorithm.

Automatic feature segmentation is carried before multi-view registration. Automatic segmentation techniques of echocardiographic images face a number of challenges due to poor contrast, high-level speckle noise, weak endocardial boundaries and small boundary gaps. Recently, promising segmentation results have been obtained using methods based on deformable models. There are mainly parametric and geometric deformable models [21]. In parametric models, the evolving segmentation curve is explicitly represented. In geometric models, the evolving segmentation curve is implicitly expressed as level sets of a higher dimensional scalar function. Unlike parametric models, geometric deformable models can handle topological changes automatically and be easily extended to higher dimensional applications.

Parametric deformable models have been used in semi-automatic segmentation for both the epicardial and endocardial borders over the entire cardiac cycle [22-24]. Here, statistical models of the cardiac structure features (e.g. shape, intensity appearance and temporal information) were derived from large training data sets for segmenting endocardial boundaries [25-28]. For these methods, we note that there is significant overhead associated with providing large and appropriate training data sets and also significant efforts in setting up the point correspondences.

In clinical practice and especially in paediatric cardiology, there can be significantly topological variability associated with ventricle wall boundaries. Due to the wide variability in possible abnormal cases, it is difficult to provide significant populations for each abnormal classification. This difficulty further limits the applicability of parametric model-based approaches. More recently, geometric level set models have been developed to address these limitations. These models can handle topological changes automatically without the need for extensive parameter training. Level set methods have been used for echocardiography image segmentation [29]. Other variational level set segmentation strategies also integrate prior knowledge (shape, statistical distribution *etc*.) [30-32]. However, the use of prior information often requires off-line training. It can be tedious and expert-dependent.

Alternatively, Corsi *et al*. [33] developed a semi-automatic level set segmentation method that did not require prior knowledge. The authors applied the method to real-time 3D echocardiography images for reconstructing the left ventricle. In this study, the initial surface had to be chosen close to the boundaries of the LV chamber.

We describe a new image segmentation method that relaxes the need for accurate initialization. Here, we are proposing a two-step approach. After a rough initialization, we first use a *gradient vector flow *(GVF) *geodesic active contour *(GAC) model to move the initial contour closer to the true boundary. This is done by driving the initial contour to the true boundary using strong GVF forces [34], which is integrated in *geodesic active contour *(GAC) model [35-38]. Then, in the second step, the evolving curve is driven by image gradient for accurate segmentation. It allows for relatively simple and free initialisation of the model, while minimizing edge leaking. We also present a study of the influence of the segmentation parameters on the model. To the best of our knowledge, no similar parameter optimization is reported in publications in ultrasound image segmentation.

The performance of the 3D system is demonstrated in its ability to provide accurate volume estimates using sparse image plane sampling from multiple acoustic views. To quantify accurate measures, the validation is focused on measures taken on calibrated 3D ultrasound phantoms. However, we also provide measures from *in-vivo *cardiac data set.

## Methods

### Hardware Setup and Software Flow Chart

For acquiring 2D ultrasound images, we use an Acuson Sequoia C256 (Siemens, USA) with a 7 MHz array transducer probe 7V3C (see Figure 1). A six-degree of freedom EPOM device, the Flock of Birds (FOB) (Ascension, Burlington, VT, USA) is used to record 3D location of each 2D image. For accurate 3D reconstructions, a calibration for determining the spatial relation between the sensor and the 2D images is performed [4,39].

Figure 2 shows the system software flow chart. We start image acquisition with breath-holding and ECG gating at the standard acoustic window. The purpose of doing this is to avoid the cardiac deformation due to respiration and cyclic cardiac motion. Then, the only significant source of misregistration is due to the rigid movement of the patient. The position and orientation of the transducer associated with each acquired 2D image (640 × 480) are saved in the computer. The region of interest (ROI) is quickly outlined to reduce the computational and memory requirements. 2D+T image sequences are segmented automatically to identify the endocardial boundaries. The 3D surface of the LV is reconstructed with automated registration using segmented boundary walls. The whole software is developed in C programming language and MATLAB (MathWorks).

### Hybrid Gradient Vector Flow (GVF) Geometric Active Contour (GAC) Model

Level sets segmentation allows the development of new image segmentation methods that can adapt to complex object boundaries. We developed a new hybrid model that can deliver accurate segmentation results from relatively simple initializations.

Level sets was first introduced by Osher and Sethian [40]. The authors started with a model of a propagating front Γ(*t*) as the zero level set of a higher dimensional function: *φ*(*x*,*y,t*). Initially, we have:

φ(x,y,t=0)=±d

where ***d ***is the signed distance from point (*x*,*y*) to the boundary of an region Ω (Γ(*t*) bounds the region Ω) at t = 0. If *d *= 0, the point (*x*,*y*) is on the boundary. For points that are inside the initial boundary, *φ*(*x*,*y,t = *0) takes on negative values. For points that are outside the initial boundary, *φ*(*x*,*y,t = *0) takes on positive values.

Osher and Sethian [40] had shown that *φ*(*x*,*y,t*) evolves according to the following dynamic equation:

$$\frac{\partial \varphi }{\partial t}+\vec{F}\cdot \nabla \varphi =0$$

with the initial condition *φ*(*x*,*y*,0) = *φ*_0 _(*x*,*y*). $\vec{F}$ is the propagation velocity function on the interface. Here, only the normal component of $\vec{F}$ is needed. The unit normal vector (outward) to the level set curve is given by:

$$\vec{n}=\frac{\nabla \varphi }{\left|\nabla \varphi \right|}.$$

The evolution equation becomes

$$\frac{\partial \varphi }{\partial t}+F\cdot \left|\nabla \varphi \right|=0$$

Where ***F ***is the normal component of $\vec{F}$, given by

$$F=\vec{F}\cdot \frac{\nabla \varphi }{\left|\nabla \varphi \right|}=0$$

For image segmentation applications, the goal is to design the speed function ***F ***so as to propagate the evolving curve to the edges of the image. There are three main types of motion in curve evolution [41]. We write as:

$$F=F_{norm}+F_{curv}+F_{adv}$$

where *F*_*norm *_denotes motion in the normal direction; *F*_*curv *_denotes motion in the curvature direction, and *F*_*adv*_denotes motion due to an externally generated velocity field, independent of the front itself.

The new hybrid deformable model is given by:

$$\frac{\partial \varphi }{\partial t}=g\varepsilon \kappa \left|\nabla \varphi \right|-\left\{(1-s\left(x,y\right))\left[\beta_{1}\left(\left(u\left(x,y\right),v\left(x,y\right)\right)\cdot \nabla \varphi \right)\right]+s\left(x,y\right)\beta_{2}\nabla g\cdot \nabla \varphi \right\}$$

The first product term in the right hand side describes motion in the curvature direction, while the motion due to externally velocity is shown in the second and third terms. Here, *ε *is constant, *k *denotes the curvature and *g *is an edge function. *g *is defined as an enhanced edge indicator applied to a Gaussian smoothed image given by:

$$g\left(x,y\right)=\frac{1}{1+{\left(\frac{\left|\nabla \left(G_{\sigma }\left(x,y\right)*I\left(x,y\right)\right)\right|}{\alpha }\right)}^{2}}$$

where α is a constant strength coefficient. *g *is close to zero in regions where the gradient is high, and is approximately one in homogenous regions.

To minimize the edge leakage, the expansion term in the normal direction is excluded in this model.

To allow the deformable models to be initialized away from the object boundary, GVF vector field (*u*(*x*,*y*), ν (*x*,*y*)) is used for external driving force at the beginning. It diffuses the image gradient toward the homogenous region, allowing curve evolution in edge-free regions. It also allows for bi-directional flow that propagates the curve toward the object boundary from inside or outside the boundary. The edge indicator function *g *is also used for controlling the strength of the advection term.

Unfortunately, the GVF field can push the curve through poor edges causing edge leakage. A step function *s*(*x*,*y*) is used to control the external force. Initially, it is zero. Then, the advection force, GVF field drives the evolving curve rapidly toward the object boundary, even in a homogeneous field. When the segmentation curve is sufficiently close to the true boundary, the edge map assumes higher values. To detect when the evolving curve is approaching the target boundary, we evaluate the average of the edge map over the current zero level-set at each iteration. When the average value is above a certain threshold, we turn on the step function. The advection term is then dominated by the vector ∇g, which can be used to prevent the evolving front from "pass through" at weak boundary or small boundary gaps. We define *s*(*x*,*y*) as

$$s\left(x,y\right)=\{\begin{array}{cc} 0, & Ave_{\varphi \left(x,y,t\right)=0}\left\{f\left(x,y\right)\right\}<T_{res}\\ 1, & Ave_{\varphi \left(x,y,t\right)=0}\left\{f\left(x,y\right)\right\}\geq T_{res}, \end{array}$$

Where *f*(*x*,*y*) is edge map function defined by:

$$f(x,y)={\left(\frac{\left|\nabla (G_{\sigma }(x,y)*I(x,y))\right|}{\alpha }\right)}^{2}.$$

### Segmentation Parameter Optimization

Corsi *et al*. [33] set the parameters empirically: *α = *0.1*, β = *6*, ε *= 0.5. For the new model, a segmentation parameter optimization is also implemented.

The proposed hybrid model requires pre-setting a single threshold parameter. It is important to optimize this parameter since it can affect the performance of the segmentation method. For example, if the threshold value is too low, the hybrid method may not be able to reach the true boundary because of the relaxed initialization. For very high values, the evolving curve may pass the true boundary given as a result leakage at the edges. For that reason, it is necessary to consider all the possible values for the threshold in the optimization. In order to cover a wider range, a logarithmic sampling of the threshold values is chosen: *T*_*res *_= [5, 20, 50, 125.6, 315.5, 792.4, 1990.5, 5000].

The same logarithmic scale is also considered for the values of the *ε, β*_1, _*β*_2 _parameters. A total of 10 different values per parameter is set in the following way: *ε *= [0.1, 0.21, 0.46, 1, 2.15, 4.64, 10, 21.54, 46.42, 100] and *β*_1_= *β*_2 _= [0.6, 1.29, 2.79, 6, 12.92, 27.85, 60, 129.27, 278.50, 600].

To evaluate segmentation performance for each parameter combination, representative images are selected. Then, a simple curve which is set to be inside the ROI but far from the true boundary is provided to the algorithm as an initialization.

Two metrics are used to determine the optimal parameters: Hausdorff distance and mean absolute difference (MAD) between the manually and automatically segmented boundaries. The Hausdorff distance measures the distance between the points on two curves that differ the most, while MAD provides the mean difference between the two curves. Finally, the minimum values of both metrics determine the optimal parameters.

### Multi-view Reconstruction with Automatic Registration

Automatic registration is a required and important step for combining acquisitions between different views. Clearly, misregistration is a big problem in freehand 3D ultrasound that affects the accuracy of the reconstruction and volume estimation. In general, there are three sources that cause misregistration in freehand 3D ultrasound: (i) spatial location error of 2D image, (ii) target movement during intra-view (deformation by cardiac motion and respiration) and inter-view scans (rigid movement of the subject), and (iii) unstable probe pressure applied on the scanning surface. The first error is largely reduced by the electromagnetic interference detector in the system [10]. The misregistration due to unstable probe movement is reduced by short acquisition time (about 15 seconds) for each view acquisition. Intra-view deformation can be addressed by breath-holding and ECG gating. Rigid movement of the subject in inter-view scans causes the majority of the registration errors.

Our basic assumption for achieving automatic registration is that there is a partial overlap between the image acquisitions from different views. And we do require that the images from different views share some common features or anatomical structures, such as chamber wall surfaces. As we pointed out earlier in this paper, only voxel intensity-based registration can lead to significant errors. This is due to ultrasound-gain variation, speckle noise, and viewing artefacts. Instead of intensity-based registration, we use a feature-based geometric approach.

The basic idea is to reconstruct each view in 3D and then register the views together. The reconstructed 3D surfaces are obtained by 3D reconstruction of the 2D planes. Here, each plane is generated using the difference between two binary images. First, we generate a binary image of the segmented region that contains all of the pixels that fall inside the object of interest. The second binary image is generated by eroding the first one using a circular element of radius of 4 to 8 pixels (based on target size). The difference image captures the boundary wall. A 3D reconstruction of the 2D planes generates a 3D binary surface model.

We note that registration is possible as long as the reconstructed wall surfaces exhibit some overlap. To satisfy the partial overlap criterion, we require that at-least one of the views is a full-sweep, covering the entire object of interest. We expect that the inherent appearances of chamber wall surfaces will guarantee the existence of a unique global minimum for the registration parameters.

To reach the globally optimal value, we first apply a global registration method by using 3D Hotelling transform to construct an object-based reference volume. This is needed to avoid local minima and ensure a significant overlap between different view acquisitions. Then, we perform a higher accuracy registration using a robust, non-linear least squares algorithm to archive the optimal parameters.

**Figure 3:**
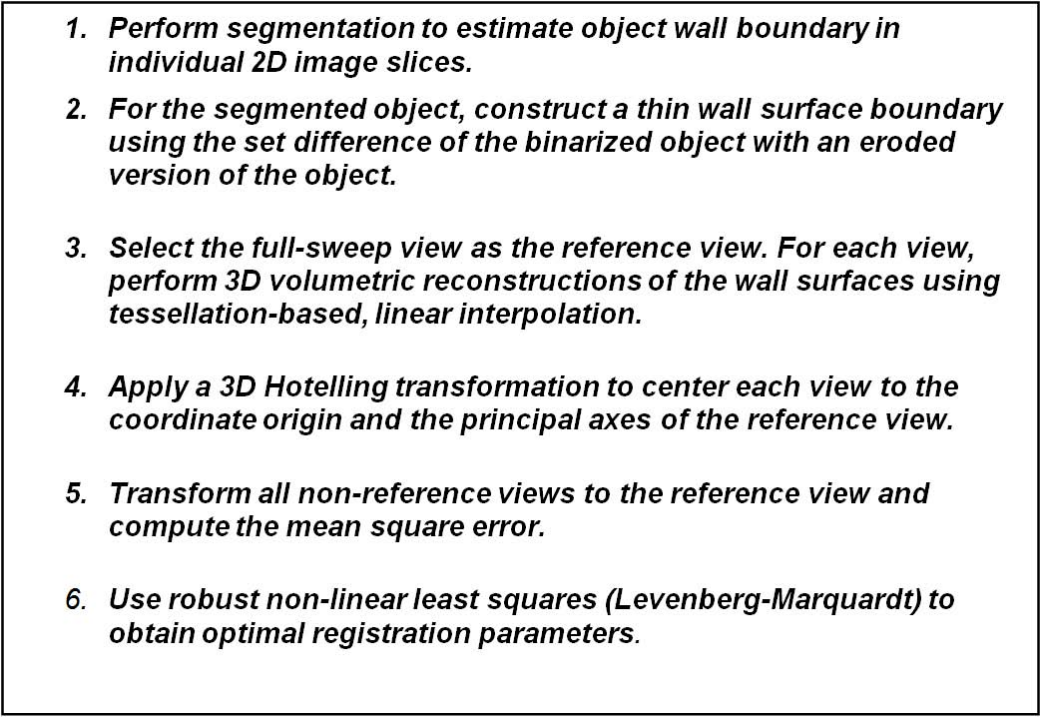
**Multiview registration algorithm**. The algorithm allows us to provide 3D reconstructions from sparse 2D images from different views. The algorithm uses a 3D Hotelling transform to perform an initial registration. This approach provides initial estimates for the optimal parameters that are later found using non-linear least squares. The parameter initialization step reduces the global optimization problem to a local optimization problem that can be solved quickly using the Levenberg-Marquardt algorithm.

The overview of the registration algorithm is given in Figure 3. Reconstructions from different views are rigidly registered to the same object-based reference volume by using 3D Hotelling transform. Then the parameters are used to initialize a more accurate registration procedure. A non-linear least square method, Levenberg-Marquardt algorithm is used to finely estimate the optimal registration parameters.

We also provide a more formal description of the procedure. Suppose we want to register two sets of image sequences acquired from two different views *V*_1 _and *V*_2_. Let *N*_*p*_*,N*_*q *_be the number of pixels inside the wall surfaces in sets *P,Q *respectively. We write:

$$\begin{array}{cc} P=\left\{{\text{p}}_{i}\right\}, & i=1,2,\cdots ,N_{p} \end{array}$$

$$\begin{array}{cc} Q=\left\{{\text{q}}_{j}\right\}, & j=1,2,\cdots ,N_{q} \end{array}$$

where **p**_*i*_,**q**_*j *_are 3D voxel coordinates from the two views:

$${\text{p}}_{i}={\left[x_{i},y_{i},z_{i}\right]}^{T}$$

$${\text{q}}_{j}={\left[x_{j},y_{j},z_{j}\right]}^{T}.$$

We then reconstruct the 3D volume with the largest number of 2D image planes (for example, view *V*_1 _) over a regular Cartesian grid, and then register the 2D image slices from the rest of the views (for example, view *V*_2_) to it.

The data points from the second view *V*_2 _are transformed using the initial transformation acquired using 3D Hotelling, denoted as *T*(**q**_*j*_). We interpolate the intensity values at *T*(**q**_*j*_) using the image points of *I*(**p**_*i*_) in 3D Cartesian grid volume. The optimal registration transformation is obtained as the one that minimizes the mean square error of the objective function:

$$f\left(P,T(Q)\right)=\frac{1}{n(T)}{\sum_{p_{i},q_{j}\in O(T)}\left[I_{R}\left(p_{i}\right)-I_{N}\left(T\left(q_{j}\right)\right)\right]}^{2}$$

where *O *is the overlapping region between the two volumes, *I*_*R *_refers to the reference 3D reconstruction, *I*_*N *_refers to the "new" 3D reconstruction to register, and ***n ***is the number of the voxels within set *O*. Once the images are registered, the 3D reconstructed volume is achieved by averaging the intensities from the different view volumes to attenuate artefacts and reduce noise.

## Results

### Data Sets and Acquisition

The system is evaluated on calibrated 3D ultrasound phantom and *in-vivo *paediatric cardiac data set. We use the standard 3D calibration phantom (Model 055, CIRS, USA) which contains two volumetric egg-shape objects that can be scanned from both top and the side windows. From each scanning window, ultrasound images can be collected at both the long-axis and short-axis views. Eight sequences of phantom image videos are used for validating single view and multi-view reconstructions. The numbers of images in each view reconstruction are shown in Table 1.

**Table 1:** Quantitative Comparison on Phantom Image Sequence Segmentation

Sequence	MAD (mm)		Hausdorff Distance (mm)	
	
	Empirical parameters	Optimal parameters	Empirical parameters	Optimal parameters
1 (40 frames)	0.9828 (σ = 0.4963)	**0.9479 (σ = 0.5209)**	3.1076 (σ = 2.0168)	**2.8946 (σ = 1.739)**

2 (40 frames)	0.6540 (σ = 0.3876)	**0.6516 (σ = 0.8613)**	1.4230 (σ = 0.8147)	**1.4156 (σ = 0.8125)**

3 (40 frames)	0.7858 (σ = 0.5158)	**0.7523 (σ = 0.2932)**	2.7191 (σ = 2.0428)	**2.500 (σ = 1.4683)**

4 (40 frames)	0.4805 (σ = 0.2927)	**0.4802 (σ = 0.2919)**	1.1981 (σ = 0.7535)	**1.1971 (σ = 0.7504)**

5 (44 frames)	**0.5105 (σ = 0.2299)**	0.5464 (σ = 0.2418)	2.1746 (σ = 1.1174)	**1.6694 (σ = 0.8680)**

6 (41 frames)	0.4687 (σ = 0.2287)	**0.4246 (σ = 0.2044)**	1.9629 (σ = 0.9913)	**1.0510 (σ = 0.5140)**

7 (44 frames)	**0.4769 (σ = 0.1677)**	0.4815 (σ = 0.1069)	1.8469 (σ = 1.1158)	**1.8016 (σ = 0.9674)**

8 (47 frames)	**0.3849 (σ = 0.1655)**	0.5153 (σ = 0.2260)	1.5914 (σ = 0.8436)	**1.2425 (σ = 0.5320)**

The best results are presented in bold-face.

While most of the current research has focused on adult cardiology, our primary focus here has been on applications in paediatric cardiology. In paediatric echocardiography, smaller heart size, higher heart rate, and more complicated cardiac anatomy make accurate 3D reconstruction even harder.

Four sequences of *in-vivo *paediatric cardiac image videos are used in the cardiac experiment. Data sets are acquired from the parasternal short-axis view and the apical long-axis view from a six year old healthy child volunteer. Breath holding (15 seconds) and ECG gating are used to minimize the deformation from respiration and cardiac motion. In each view acquisition, the transducer is moved slowly and evenly to scan the heart. Due to the standard frame rate of the frame grabber (nearly 30 frames per second), 433 images with 640 × 480 resolution are collected in 15 seconds, which is a much shorter scanning time than the acquisition time reported by Ye *et al*.[7]. The subject does not have to remain still during the time it takes to switch to a different acoustic window. The image acquisition procedure for 3D is done exactly in the same way as the regular routine echocardiography examination at the hospital.

### Segmentation

**Figure 4:**
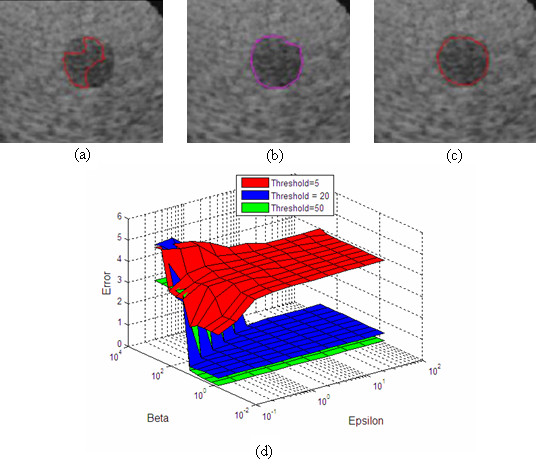
**Segmentation parameter optimization for the calibrated phantom image (Hausdorff criterion)**. (a) Best segmentation result for threshold value of *T*_*res *_= 5, b) Best segmentation result for *T*_*res *_= 20, (c) Best segmentation result for *T*_*res *_= 50, (d) Hausdorff distance surface as a function of *ε *and *β =β*_1 _*= β*_2 _for 3 different thresholds: *T*_*res *_= 5 with minimum error value of 2.25 (*ε *= 0.1,*β *= 12.92), *T*_*res *_= 20with minimum error value of 0.38 (*ε *= 4.64,*β *= 600), *T*_*res *_= 50 with minimum error value of 0.09 (*ε *= 0.1,*β = *0.6).

**Figure 5:**
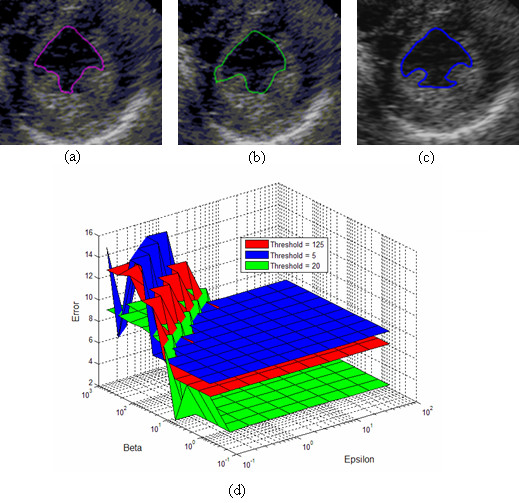
**Segmentation parameter optimization for a cardiac image (Hausdorff criterion)**. **(a) **Best segmentation for threshold value *T*_*res *_= 5, (b) Best segmentation result for *T*_*res *_= 125, (c) Best segmentation result for *T*_*res *_= 20, (d) Hausdorff distance as a function of *ε *and *β =β*_1 _*= β*_2 _for 3 different thresholds: *T*_*res *_= 5 with minimum error value of 7.32 (*ε *= 0.464,*β *= 1.29), *T*_*res *_= 125 with minimum error value of 6.14 (*ε *= 0.1,*β *= 0.6), *T*_*res *_= 20 with minimum error value of 2.30 (*ε *= 0.1,*β *= 1.29).

Two typical ultrasound images (a phantom image and a cardiac image) for estimating the optimal segmentation parameters are selected. Figures 4 and 5 show the images used in the optimization and their results. Hausdorff distance and MAD are used to measure the difference between the manual and automatic segmentation boundaries. Similar results are obtained when optimising for the minimal Hausdorff distance or MAD. In what follows, we present and discuss results for the Hausdorff distance.

For the phantom image, the minimum Hausdorff distance is achieved for: ε = 0.1, β_1 _= β_2 _= 0.6, *T*_*res *_≥ 50 (see Figure 4). From the plots of Figure 4, it is interesting to note that the optimization level is relatively flat within a certain region of the *ε*-*β *plane. In particular, the heuristically derived point given by ε = 0.8, β_1 _= β_2 _= 6, *T*_*res *_= 50 (used in our earlier investigations) gives essentially equivalent performance to the optimal line given by ε = 0.1, β_1 _= β_2 _= 0.6, *T*_*res *_≥ 50. To minimize computational complexity, we use the point given *T*_*res *_= 50. It allows for the evolving front to converge quickly under GVF force to the region around the boundary, before it allows for local gradient to slowly fine-tune the final result. Here, the optimal results require that *β *values be at-least six times larger than the *ε *value where *ε *lies in the interval of [0.1, 100].

For the cardiac image, the optimal segmentation parameters are given by: *ε = *0.1, *β*_1 _= *β*_2 _= 1.29 and 5 <*T*_*res *_< 125 (see Figure 5). As compared to the phantom image, the actual cardiac images require lower value of *T*_*res*_, and much larger values for the *β *parameters. For optimal results, we require a threshold value of 20 with *β *values to be at-least ten times larger than *ε *where *ε *lies in the interval of [0.1 100].

**Figure 6:**
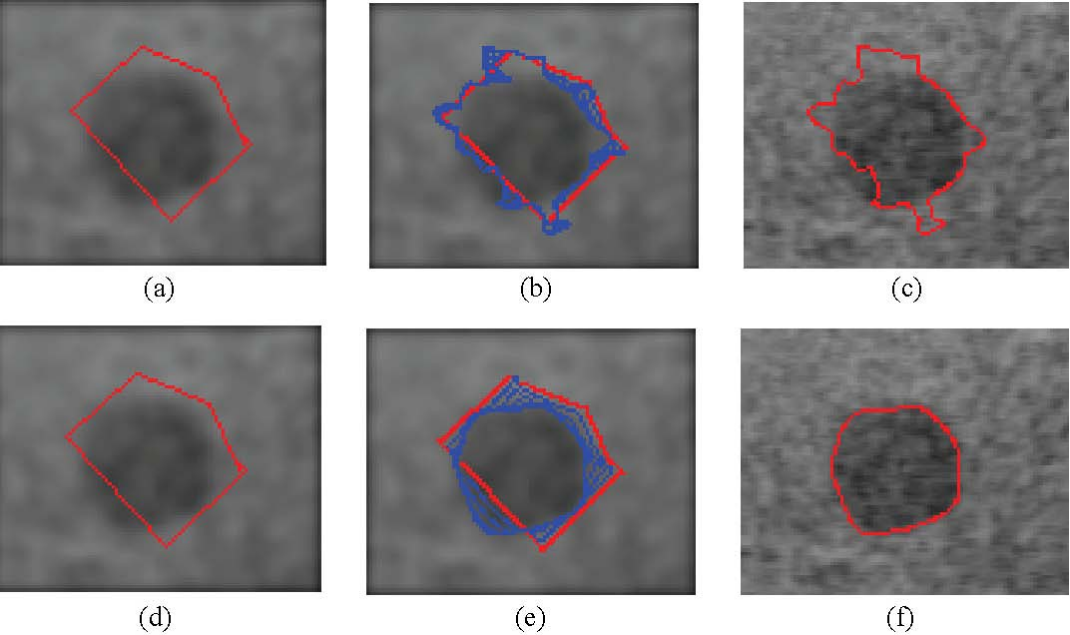
**Comparison between Corsi's method and the new hybrid GVF GAC model**. (a) Gaussian smoothed image (*σ*_*G *_= 2) with the initial curve, (b) Curve evolution using Corsi's method (c) Result of Corsi's method; (d) Same initialization as in (a), (e) Curve evolution using the new, hybrid method, (f) Result of the new method.

An example of the robustness of the new hybrid model is shown in Figure 6. In this phantom image example, we have a comparison between the new hybrid method and Corsi's method. In both cases, the initial curve was set to the pentagon shown in Figures 6a,d. The new hybrid method produces an accurate segmentation result (Figure 6f) as opposed to the failed segmentation result of Figure 6c. In this case, the GVF field of the new, hybrid method has helped push the initial segmentation curve to the true boundary. Here, the strength of the GVF field has contributed to the robustness of the method.

**Figure 7:**
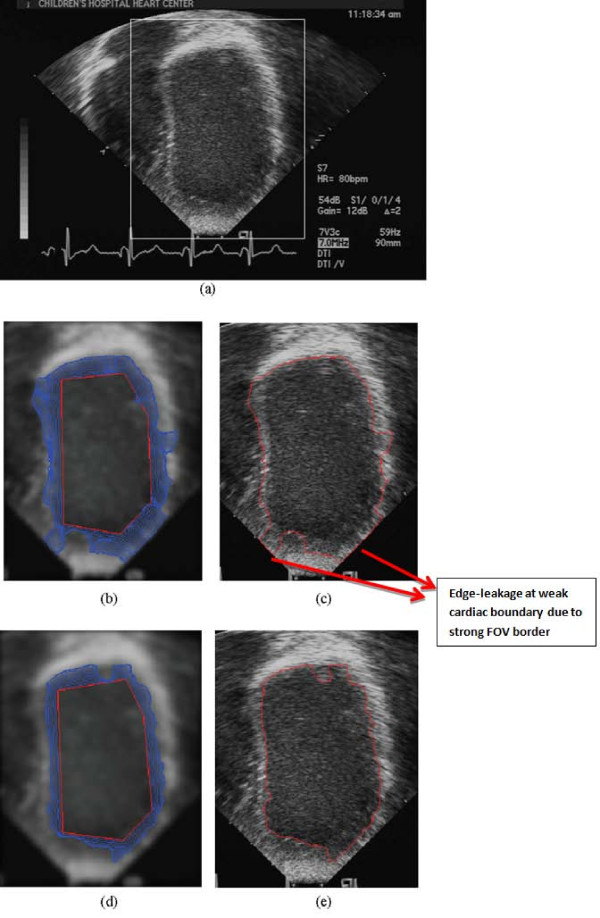
**Comparison of Paragios' method and the new hybrid GVF GAC model**. Prior to segmentation, the images were smoothed with a Gaussian window with *σ*_*G *_= 3 (a) A apical long-axis image, (b) Curve evolution using Paragios' method, (c) Result of Paragios' method (d) Curve evolution using the new hybrid method, and (e) Result of the new method.

Comparative results between the new, hybrid method and Paragios' approach are shown in Figure 7. The image is from an apical long axis view of a child's LV. Here, we note the weak edge on the left and right bottom side of the endocardial boundary compared to the strong field of view border. Figure 7e demonstrates that the new segmentation method accurately converges to the endocardial boundary. On the other hand, results from Paragios' method [38], presented in Figure 7c, appear to suffer from edge leakage, the evolving curve passing over the cardiac boundaries, towards the border of the field of view of the image. In the new, hybrid method, this problem is solved since the proximity to the cardiac boundary was detected, and the external force switched from the strong GVF to the edge-map gradient.

We evaluate the performance of the new segmentation method on eight sets of phantom image (2D+T) sequences. To initialize the segmentation, we require that the users provide an initial curve from a frame in the middle of the image sequence. The segmentation procedure then proceeds to automatically segment images before and after the middle image. For each frame, segmentation is initialized by using the segmented curve from the previous frame, allowing for a quick convergence and accurate segmentation.

In order to discuss the effects of the segmentation parameters, we present results for both optimized and non-optimized (empirical) segmentation. For the non-optimized segmentation, we set the parameters empirically, after a few experiments with a couple of training images (*ε *= 0.8, *β*_1 = _*β*_2 _= 6, *T*_*res *_= 50). For the optimized results, we follow the optimization method that we described in the Methods Section.

Table 1 shows that the optimal parameters gave the lowest Hausdorff distance errors in all cases. For the MAD, the optimized approach gives the best results in the majority of the cases. On average, the MAD stays below 1mm while the maximum segmentation error stays below 3mm (Hausdorff distance). It is interesting to note that there is more consistency in the optimized approach, in the sense that the standard deviations of the Hausdorff distances is found to be remain lower than the empirical approach.

**Figure 8:**
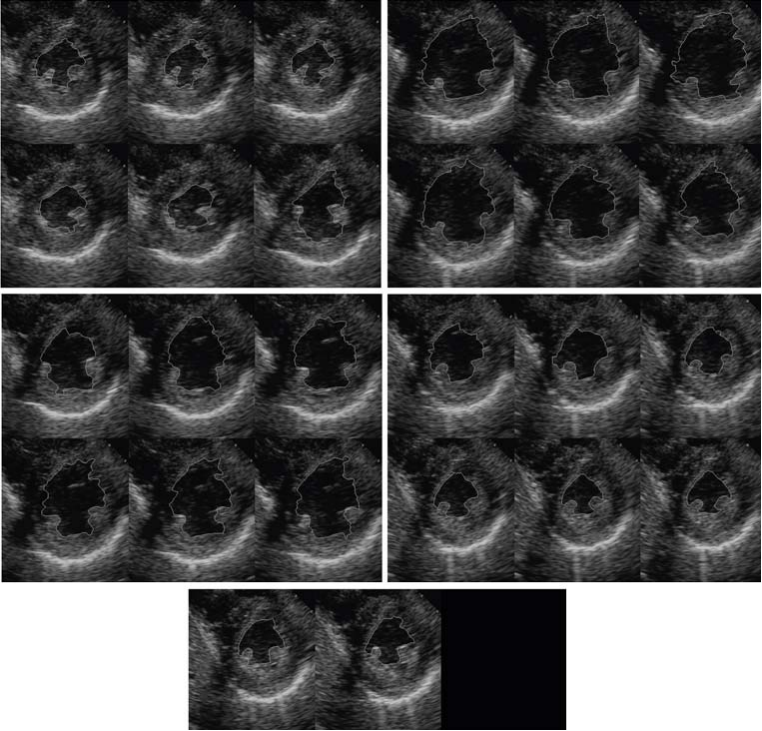
**Parasternal short axis sequence segmentation using the new, hybrid method**.

For the echocardiography images, both of the sequences from the parasternal and apical views have 26 frames for a full cardiac cycle. We present qualitative results for parasternal view sequence in Figure 8 and quantitative results in Table 2. We note significant improvements of the optimized approach for the apical image sequence. The results for the parasternal case do not show the same levels of improvements. We attribute the improvements to the relative regularity of the images obtained from the apical sequence. This regularity is missing from the images in the parasternal sequence. Thus, the regularity in the apical sequence helps the optimization process.

**Table 2:** Quantitative results on echocardiography sequence segmentation.

Echocardiograpy Sequences	MAD (mm)		Hausdorff Distance (mm)	
	
	Empirical parameters	Optimal parameters	Empirical parameters	Optimal parameters
Parasternal (26 frames)	0.794 (σ = 0.272)	**0.775 (σ = 0.276)**	3.656 (σ = 1.210)	**3.610 (σ = 1.401)**

Apical (26 frames)	1.267 (σ = 0.266)	**0.911 (σ = 0.171)**	4.261 (σ = 0.899)	**2.966 (σ = 1.127)**

The best results are presented in bold-face.

### Multi-view Reconstruction with Registration

We provide validation in terms of volume measures for the calibrated phantom and left ventricle in paediatric echocardiography. We observe a relation between the relative volume error and the number of image frames and the number of views in calibrated phantom.

**Figure 9:**
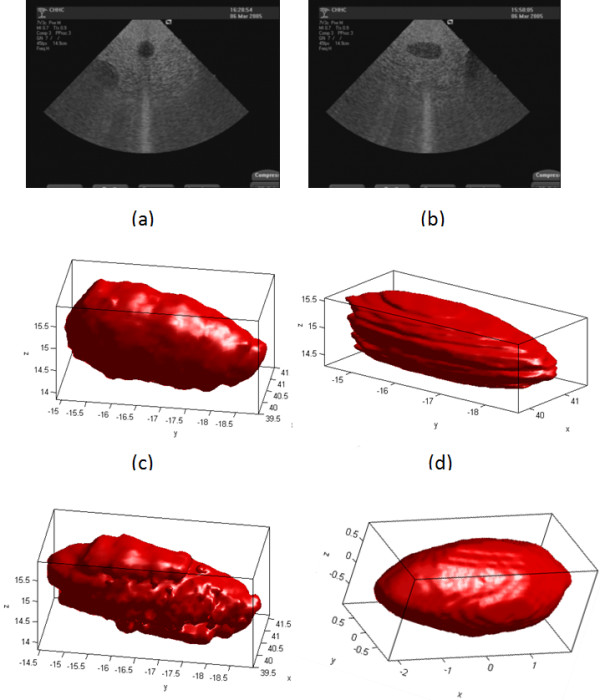
**Multi-view reconstruction with registration for the calibrated phantom**. (a) short-axis view image, (b) long-axis view image, (c) short-axis view reconstruction, (d) long-axis view reconstruction, (e) two-view reconstruction without registration, and (f) two-view reconstruction with automatic registration.

An example of the phantom image from the short-axis and long-axis views is shown in Figure 9a-b. Forty image slices were used with a region of interest (ROI) of 70 × 70 pixels in the short-axis view and 40 images with 110 × 60 pixels ROI in the long-axis view. Single view reconstructions are shown in Figure 9c-d respectively. We note that the long-axis view reconstruction appears to miss some of the data at the extremes of the z-axis. Such extreme edge artifacts are clearly unavoidable in single view reconstruction. The two-view reconstruction without registration is shown in Figure 9e. Without multi-view registration, the accurate volume, even the 3D shape of the object could not be obtained correctly. The reconstruction with volumetric registration is shown in Figure 9f. The improvement is significant.

Table 3 presents volume estimates from single-view, two-view, and three-view reconstructions for phantom. We note that there are large errors in some single view reconstructions, while two-view and three-view reconstructions consistently gave significantly smaller errors. Overall, the multi-view reconstruction volume error remained below 3%.

**Table 3:** Phantom volume measures and relative error for one-view, two-view, and three-view reconstructions.

**Small Egg (7.2cc)**	**View** **Number**	**View Locations**	**Frame** **Number**	**Volume** **Estimate**	**Relative** **Error**
	
	1 view	Top window, short-axis	41	7.4218cc	3.08%
		
		Top window, long-axis	44	5.1246cc	-28.83%
		
		Side window, short-axis	47	7.4441cc	3.39%
		
		Side window, long-axis	44	4.7494cc	-34.04%
	
	2 views	Top window	Short-axis 21 Long-axis 22	7.4072cc	2.88%
		
		Side window	Short-axis 24 Long-axis 22	7.3697cc	2.36%
	
	3 views	Top & Side windows	Top window, short-axis 21 Top window, long-axis 22 Side window, long-axis 22	7.0045cc	-2.72%

**Figure 10:**
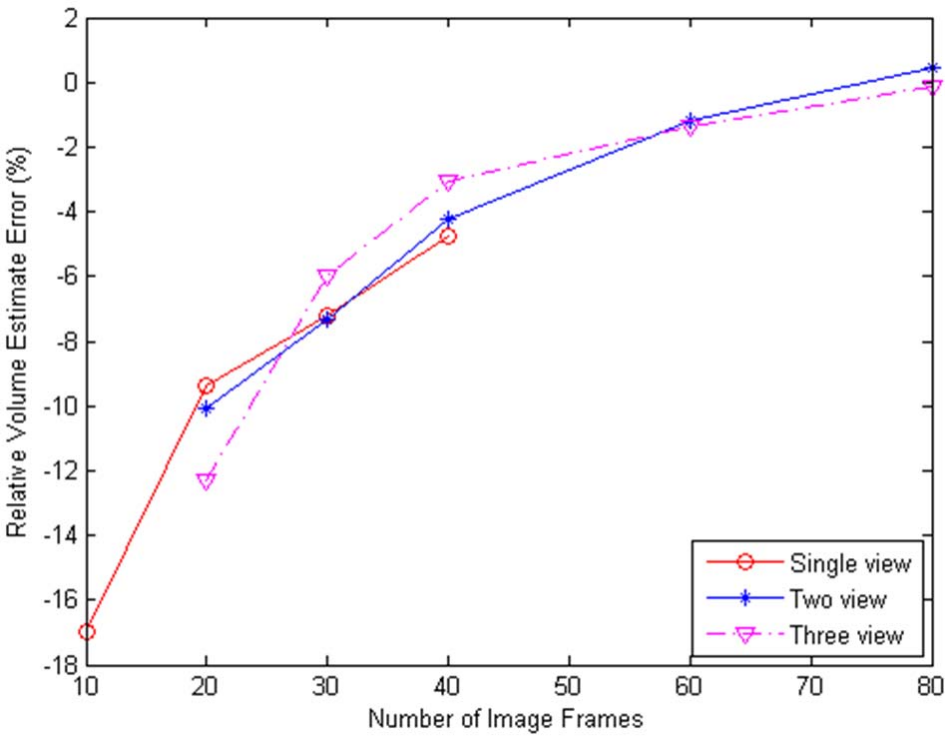
**Graph of the relation between volume relative error and the number of views and the number of image frames**.

Figure 10 presents the relation between the relative reconstruction error and the number of views and the number of image frame. We performed single view reconstructions using 10, 20, 30 and 40 image frames respectively, and also performed two-view and three view reconstructions using 20, 30, 40, 60 and 80 frames from the different views.

We note that two-view and three-view reconstructions that combine all available images gave the most accurate reconstructions. In fact, registering image planes from different views had obtained approximately the same error as reconstructions with the same number of image planes from a single view. This shows that automatic registration of multi-view sparse images was successful. Further discussion of this figure is given in Discussion section.

Comparative reconstructions using automatic and manual segmentation are given in Table 4. In most cases, the optimized segmentation gave significantly better results than the empirical segmentation. In one of the two-view reconstructions, the optimized segmentation approach gave nearly perfect results at 0.06%. In all cases, the optimized segmentation error remained below 5%.

**Table 4:** Volume measures using two-view reconstruction with automatic segmentation and manual segmentation.

Two-view reconstruction	Manual Segmentation		Automatic Segmentation with empirical parameters		Automatic Segmentation with Optimal parameters	
	
	Volume Estimate	Relative error	Volume Estimate	Relative error	Volume Estimate	Relative error
Top window: long-axis and short-axis	7.4072cc	2.88%	7.6853cc	6.74%	**7.1395cc**	**-0.84%**

Side window: long-axis and short-axis	7.3697cc	2.36%	7.4599cc	3.61%	**7.4287cc**	**3.18%**

Top window short-axis and side window long-axis	7.2431cc	0.60%	7.0314cc	-2.34%	**7.2040cc**	**0.06%**

Top window long-axis and side window short-axis	6.8225cc	-5.24%	**6.9608cc**	**-3.32%**	7.5376cc	4.69%

Calibrated phantom object volume is 7.2cc. The best results are presented in bold-face.

**Figure 11:**
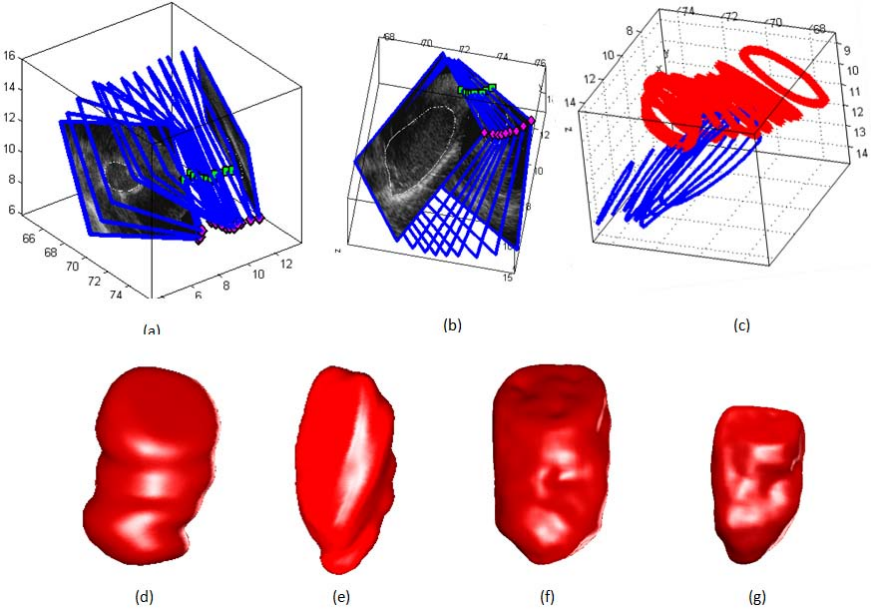
**Two view 3D reconstruction using *in-vivo *cardiac images**. (a) parasternal short-axis view image slices, (b) apical-long axis view image slices, (c) 3D spatial locations of the acquisitions. Limited partial overlaps exist between the different views, (d) parasternal view reconstruction at ED phase, (e) apical view reconstruction at ED phase. (f) two-view reconstruction at ED phase, (g) two-view reconstructions at ES phase.

Figure 11 shows an example of in-vivo cardiac data for the end of diastole (ED) and end of systole (ES) phases. For the ED phase, fourteen images are usable with a region of interest (ROI) of 240 × 210 pixels in the parasternal short-axis view (Figure 11a). In the apical long-axis view, nine images are usable with an ROI of 240 × 220 pixels (Figure 11b). Two scan volumes from the different views overlapped over a very small region in 3D space (Figure 11c). The single view reconstructions are shown in Figurs 11d-e. The two-view reconstruction with registration at ED phase is shown in Figure 11f. It is clear that the two-view reconstruction combined the information from the two different views and gave a more comprehensive and better result.

The quantitative results is shown in Table 5, the two-view reconstruction is in much better agreement with the echocardiography specialist measures, with a relative error of 2.8% in ED phase (see [11] for clinical measure approach). Here, we use the same number of image planes and ROI-sizes for the ES phase and had similar results for the two-view reconstruction at the ES phase. The two-view reconstruction of Figure 11g gave a relative error of 8.9% at the ES phase, which is significantly lower than that from single-view reconstructions.

**Table 5:** Left ventricular volume measures and relative error at ED and ES phases

Cardiac Phase		Parasternal short-axis Recons	Apical long-axis Recons	Two-view Recons	Echocardiography Specialist
	
End-of- diastole	Volume	47.64 cc	31.24 cc	46.16 cc	44.90 cc
	Relative error	6.1%	-30.4%	2.8%	
	
End-of-systole	Volume	20.28 cc	13.87 cc	19.71 cc	18.10 cc

	Relative error	12.0%	-23.4%	8.9%	

## Discussion

Our experimental results on calibrated phantom and *in-vivo *paediatric echocardiography show that the new freehand 3D system can be used to provide accurate object volume measures using sparse 2D images. The new deformable model uses GVF to provide quick convergence to the true boundaries that are relatively insensitive to the initial curves. Also, it is more robust to speckle noise, poor edges and small boundary gaps that are often found in ultrasound images.

One of the most important factors that affect the performance of the algorithm is the threshold and parameters values in the deformable model. We investigated optimal parameter regions for the new approach. Volume measures from multi-view reconstructions are found to be consistently and significantly more accurate than those from the commonly used, single view reconstructions. More importantly, we have presented the relation between percentage error and the number of views and the number of planes for 3D phantom (see Figure 10).

There are a number of important observations that can be made from Figure 10. First, we note that reconstruction error is always reduced with the increasing number of image planes. Second, we note that when the number of images is 20, single view reconstruction volume has the smallest error. The number of image planes collected from each view in two-view acquisition is 10, while in three-view acquisition it is approximately 6 or 7. The image planes in each view are too sparse to register accurately in multi-view reconstruction in this case. When the number of images is 30 and 40 (40 is the maximal number of images from single view in the experiment), two-view and three-view reconstructions consistently give more accurate results than single view reconstruction. This shows that multi-view reconstruction with volumetric registration is successful. The new registration method allows us to register different views with sparse image planes and no manual initialization. Two-view and three-view reconstructions that combine images give the most accurate reconstructions when the total number of image frames keeps the same as that in single view acquisition. Since this phantom example had no viewing obstructions, we would clearly expect that multi-view systems will provide even more dramatic improvements when obstruction is an issue. Last, we note that the graph of Figure 10 may be used as a general guide for the required number of image planes for achieving desired reconstruction accuracy in our system. As an example, for multi-view reconstructions, we would require about 40 registered image planes for a relative accuracy of 5% or less. For clinical imaging, this information can be used to determine the minimum amount of acquisition time. This is clearly a topic of special concern in clinical imaging applications.

We also discuss our results for *in vivo *cardiac data. Here, using the new registration method, we have reduced the required image acquisition time to 15 seconds per view. We have also found that small children can hold their breath during the required 15-second period. No requirement of stay still for the small children between different view acquisitions. Multi-view reconstructions only require partial overlaps from the different views and we have achieved good registration results despite the use of very small numbers of 2D planes. Qualitative and quantitative results in Figure 11 and Table 5 show significant improvements in two-view reconstructions versus any of the single-view reconstructions.

On the other hand, registration performance can clearly be affected by the segmentation results. We found that in our experiments, the significant overlaps may be required for complex endocardial boundaries in echocardiography.

In this paper, we combine 3D reconstructions from different views using simple averaging. Alternative fusion strategies have been reported [13,20]. A weighted averaging approach may be used to reduce view-dependent artifacts, further reduce noise, and emphasize anatomical structures of interest.

## Conclusions

We have presented a new freehand ultrasound system that allows 3D reconstructions from sparse sampling geometries and multiple views. The system allows the imaging specialist to optimize view selection as done in routine 2D echocardiography. The utility of the system has been carefully measured on a 3D calibrated ultrasound phantom and also in cardiology clinical settings.

More specifically, 3D performance of the proposed system has greatly benefited from a new hybrid method for image segmentation, and a new coarse-to-fine registration method. The proposed hybrid segmentation method gives optimal results over a wide range of parameters. Unlike previous methods, the proposed registration method does not require good spatial alignment between different views, and it also does not require manual initialization. Registration is performed in a fully automatic mode. On the calibrated phantom example, our approach shows that reconstruction accuracy always increases as a function of the number of views and the number of acquired 2D planes.

Extensive clinical validation is still required before the system can be employed in standard practice. We recognize that the lack of large clinical datasets is a limitation of the current study. Having said this though, it is also clear that 3D reconstruction accuracy is very hard to establish on real, clinical datasets. To address this, it is common practice to report results on calibrated 3D ultrasound phantoms, as we did in this paper.

## Competing interests

The authors declare that they have no competing interests.

## Authors' contributions

HY carried out most of the investigation, design and development. MSP conceived of the project, participated and advised in the design and coordination with the clinicians. CA participated in study design, especially parameter optimization. MBG participated in the study design and clinical image collection, provided clinical guidance. All authors read and approved the final manuscript.
